# Interactive effects of agricultural landscape heterogeneity and weather conditions on breeding density and reproductive success of a diurnal raptor

**DOI:** 10.1002/ece3.11155

**Published:** 2024-03-12

**Authors:** Inga Kujala, Hannu Pöysä, Erkki Korpimäki

**Affiliations:** ^1^ Department of Environmental and Biological Sciences University of Eastern Finland Joensuu Finland; ^2^ Section of Ecology, Department of Biology University of Turku Turku Finland

**Keywords:** agroecosystems, Eurasian kestrel, food availability, habitat heterogeneity, reproductive success, weather effects

## Abstract

Agricultural intensification and climate change are serious threats toward animal populations worldwide. Agricultural intensification reduces the heterogeneity of agricultural habitats by diminishing crop variation and destroying microhabitats, such as small woody features, whereas the effects of climate change range from the growing frequency of weather extremes to disrupted prey–predator dynamics. We collected long‐term ringing data from a population of Eurasian kestrels (*Falco tinnunculus*) located amidst agricultural areas in western Finland during 1985–2021, which we combined with density indices of their main prey species (voles), spatial data consisting of land cover classification of kestrel territories, and weather data, to study the effects of different environmental drivers on breeding density and success. We found that the density of inhabited nests rose with vole abundance and springtime snow depth, with the overall trend of population growth being stronger in areas with more heterogeneous landscapes. Clutch size was influenced negatively by the age of male parent and landscape heterogeneity, and positively by vole abundance, with rainfall having a negative influence conditional to other variables. Likewise, the number of produced fledglings was affected by male age, but it was additionally positively associated with landscape heterogeneity and its interaction with rainfall, with greater fledgling output in heterogeneous landscapes during high precipitation. The discrepancy between factors predicting large clutches and high numbers of fledglings suggests that while kestrels do not prefer heterogeneous landscapes when prospecting for territories, heterogeneous habitats provide better circumstances for foraging during the nestling period, which ensures nestling survival, particularly during adverse environmental conditions. Therefore, breeding in areas under intense agricultural use is more suboptimal to kestrels than their territory preferences would indicate. As changing climate may reduce prey availability and heighten the probability of weather extremities, agricultural intensification may lead to weaker reproductive success in densely populated farmland habitats.

## Graphical abstract

The article describes a study that combines long‐term ringing data of Eurasian kestrels (*Falco tinnunculus*), spatial data with land cover classification, density indices of main prey species, and weather data to study the effects of different environmental parameters on breeding density and success of an avian predator in agricultural habitats. We found a discrepancy between factors predicting large clutches and high numbers of surviving fledglings, suggesting that while kestrels do not find benefits to landscape heterogeneity when prospecting for territories, heterogeneous habitats provide better circumstances for foraging during the nestling period, which ensures nestling survival, particularly during adverse environmental conditions. Therefore, breeding in areas under intense agricultural use may be suboptimal to kestrels, particularly under the effects of climate change.

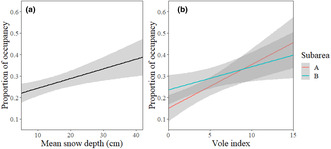

## 1 INTRODUCTION

Agricultural intensification has rapidly modified farmlands into more homogeneous environments as contiguous agricultural lots have grown bigger, crop rotations have been simplified, efficient irrigation systems have been introduced, and temporal variation in crops have been reduced, causing a recession in biodiversity among agricultural habitats (Donald et al., 2001; Filloy & Bellocq, 2007; Frenzel et al., 2016; Fuller et al., 1995; Piha et al., 2007; Rigal et al., 2023). The deprivation of semi‐natural habitats such as the margins of fields and open ditches, woodlots, and isolated mature trees in modern agricultural environments has been connected to population declines in agricultural ecosystems (e.g. Fischer et al., 2010; Gaston et al., 2003). Maintaining landscape heterogeneity in these environments has been proposed to benefit agroecosystems by providing suitable habitats to a wider assemblage of species (Benton et al., 2003; de Zwaan et al., 2022; Duelli & Obrist, 2003; Wesemeyer et al., 2023), many of which are currently estimated to be at risk (Butet et al., 2010; De Frutos et al., 2015; Kamp et al., 2021; Murgatroyd et al., 2016), while simultaneously improving ecosystem services relevant to food production such as pollination and natural pest control (de Zwaan et al., 2022).

Anthropogenic habitat alterations may disturb the process of habitat selection in a way that has fitness consequences for habitat‐selecting individuals. According to the optimal foraging theory, microhabitats abundant with food resources should be preferred in territory selection to optimize the energy and time expenditure of foraging (Pyke et al., 1977; Stephens & Krebs, 1986). Subsequently, the ideal dominance distribution (IDD) model postulates that high‐quality territories are sought after by many and eventually occupied by the fittest individuals, whereas low‐quality territories are claimed progressively only after better options have been depleted (Fretwell & Lucas, 1969; Sergio et al., 2007). Among farmland territories, heterogeneous semi‐natural patches provide greater opportunities for foraging and nesting than homogeneous expanses of fields and pastures (Mirski & Väli, 2021; Moore et al., 2003; Morelli, 2013; Šálek et al., 2020). However, in areas heavily altered by intense agriculture, remnants of these semi‐natural landscape features may not provide reliable cues of habitat quality, which may result in an outcome that violates both the optimal foraging theory and the ideal dominance distribution model. Indeed, farmland species have been reported to seek outright unsuitable habitats due to misleading cues luring them into areas under intensive agricultural activity (Arroyo et al., 2003; Grüebler et al., 2008; Schekkerman et al., 2009).

Concurrent to the advancement of agricultural intensification, species of agricultural areas are currently facing rapid environmental changes under the effects of climate change. In avian populations, negative responses to the changing climate have been documented globally (e.g. Both et al., 2006; Stephens et al., 2016), and in birds of prey, climate change has been proposed to influence many long‐term population declines that have occurred in the past decades (Catry et al., 2011; Fernández‐Bellon et al., 2020; Schmidt et al., 2012; Steenhof & Peterson, 2009). As birds of prey generally possess long life history cycles while their prey species with short generation times are capable of rapid responses to environmental shifts, they are particularly susceptible to drastic ecological changes (Meserve et al., 2003; Newton, 1979; Sergio et al., 2008). The reproductive success of raptors has been shown to be vulnerable to adverse weather conditions, such as rainfall curtailing foraging opportunities, late‐persisting snow cover reducing the availability of prey, and high wind velocity hampering prey detection (Kämpfer et al., 2022; Kostrzewa & Kostrzewa, 1990; Lehikoinen et al., 2009; Selås, 2001), making them particularly susceptible to the weather extremes anticipated to become more frequent due to climate change (Mann et al., 2017).

As anthropogenic processes have transformed most of natural grassland habitats into agricultural land, grassland specialists are largely constrained to habitats under extensive land use (Scholtz & Twidwell, 2022). Agriculture has been estimated as one of the most pressing threats to vulnerable raptor species, and under the effects of agricultural intensification and climate change, open‐area birds of prey are caught in a crossfire of ecological stress originating from anthropogenic activity (Carrete et al., 2009; McClure et al., 2018). Regarding raptors, the combined or interactive effects of these two major forces (climate change and agricultural intensification), however, have not been studied extensively on an individual level with long‐term data spanning multiple decades.

The Eurasian kestrel (*Falco tinnunculus*, hereafter kestrel) is a small, diurnal bird of prey inhabiting various open landscapes, of which arable land and agricultural fields are the most populated in Finland (Valkama & Korpimäki, 1999). While its European populations plummeted in the late 1950s and 1960s due to the concentration of pesticides (reviewed by Village, 1990), breeding densities were restored to a robust state by the end of the 1980s in Finland alongside the popularizing of suitable nest‐boxes (Korpimäki, 1983; Saurola, 2008). After their initial recovery from the bottleneck, breeding populations over all of Europe have decreased by 40–50% from 1980 (Constantini & Dell'Omo, 2020); in Finland, however, the kestrel population remains in a state of moderate growth (Honkala et al., 2021). Breeding kestrel populations established in nest‐boxes have been found to function as population sources, producing more surviving fledglings than required to replace perished breeding individuals from the local stock and supporting sink‐type populations via dispersal (Fay et al., 2019). Therefore, the fecundity of kestrels breeding in nest‐boxes might influence the stability of a larger scale of populations, which is why it is crucial to investigate how reproductive success and the pattern of demographic fluctuations are affected by anthropogenic activities in these source populations. Even as pesticide use has grown more regulated and generally safer for wildlife, open‐area predators face various agriculture‐originated threats, including degraded habitat quality, harvester‐induced nestling mortality, and rodenticide poisoning, which have likely contributed to population declines among several species (Amar & Redpath, 2005; Arroyo et al., 2003; Coeurdassier et al., 2014; Tella et al., 1998).

Small mammals, particularly the voles of the genus *Microtus*, are the cornerstone of the kestrel diet (Korpimäki, 1985; Village, 1990). Pastures, margins of agricultural fields, hedgerows, recently planted forests, and other uncropped habitats provide both cover and foraging prospects and are therefore frequented by prey animals of kestrels (Moore et al., 2003; Šálek et al., 2020). Despite the presumed advantages of heterogeneous (smaller fields intertwined by varied landscape such as woody features, semi‐natural patches, and open ditches) over homogeneous (heavily dominated by cropland, with maximized field surface and underground drainage) agricultural habitats under intensive anthropogenic influence, kestrels do not show a clear preference for nest sites surrounded by diverse landscape in terms of occupancy rates and the order of territory selection (Navarro‐López & Fargallo, 2015; Sumasgutner et al., 2019). The kestrels of Northern Europe are long‐distance migrants, initiating breeding just shortly after their springtime arrival from their wintering grounds in Southern Europe and North Africa (Palokangas et al., 1992; Village, 1990). Kestrels prospecting in spring are likely attracted to territories composed of large continuous agricultural fields which appear the most lucrative for foraging as they are uncovered by snow earlier than areas with taller vegetation (Koivusalo & Kokkonen, 2002; Sonerud, 1986). Thus, we hypothesize that homogeneous farmland might present an ecological trap (sensu Schlaepfer et al., 2002) to kestrels, misdirecting them toward less favorable habitats heavily altered by agriculture.

Availability of food resources dictates both the initial investment in breeding and its ultimate output for most birds of prey, and the kestrel is no exception (Korpimäki & Wiehn, 1998; Newton, 1979). Pre‐laying food abundance is a primary determinant of clutch size in kestrels, mirroring the quality of both the territory and the male parent, who is the main provider from prior to egg‐laying to the post‐fledging period (Korpimäki & Wiehn, 1998; Tolonen & Korpimäki, 1994). Furthermore, an abundance of main prey directs the distribution and breeding population densities of kestrels, as they nomadically disperse in search of ample prey populations (Vasko et al., 2011). In Northern Europe, kestrels prey primarily on *Microtus* voles that follow a high‐amplitude three‐year cycle in their population oscillations maintained by predation pressure (Korpimäki & Krebs, 1996). With diminishing population peaks, these oscillations have become less regular (Avotins et al., 2023; Hanski & Henttonen, 1996; Korpela et al., 2013), likely influenced by the shift in ecological dynamics caused by climate change (Cornulier et al., 2013; Ims et al., 2008). As climate change dampens the amplitudes of vole population cycles, previously established cues for habitat selection and investment in breeding might not align with a true abundance of prey animals.

Here, we explore the following questions: (1) do abiotic weather effects, territory land cover heterogeneity, and food availability modify clutch size, fledgling production, and breeding population densities of kestrels; (2) do interactions between habitat composition and weather affect these breeding parameters, with heterogeneous habitats enabling enhanced reproductive success under adverse environmental conditions; (3) are large clutches and high numbers of fledglings predicted by different environmental factors, particularly in regard to landscape heterogeneity, which would indicate that there is a discrepancy between the expected and genuine territory quality, the former being reflected in clutch size and the latter in realized reproductive output?

## 2 MATERIALS AND METHODS

### 2.1 Study area and data collection

**FIGURE 1:**
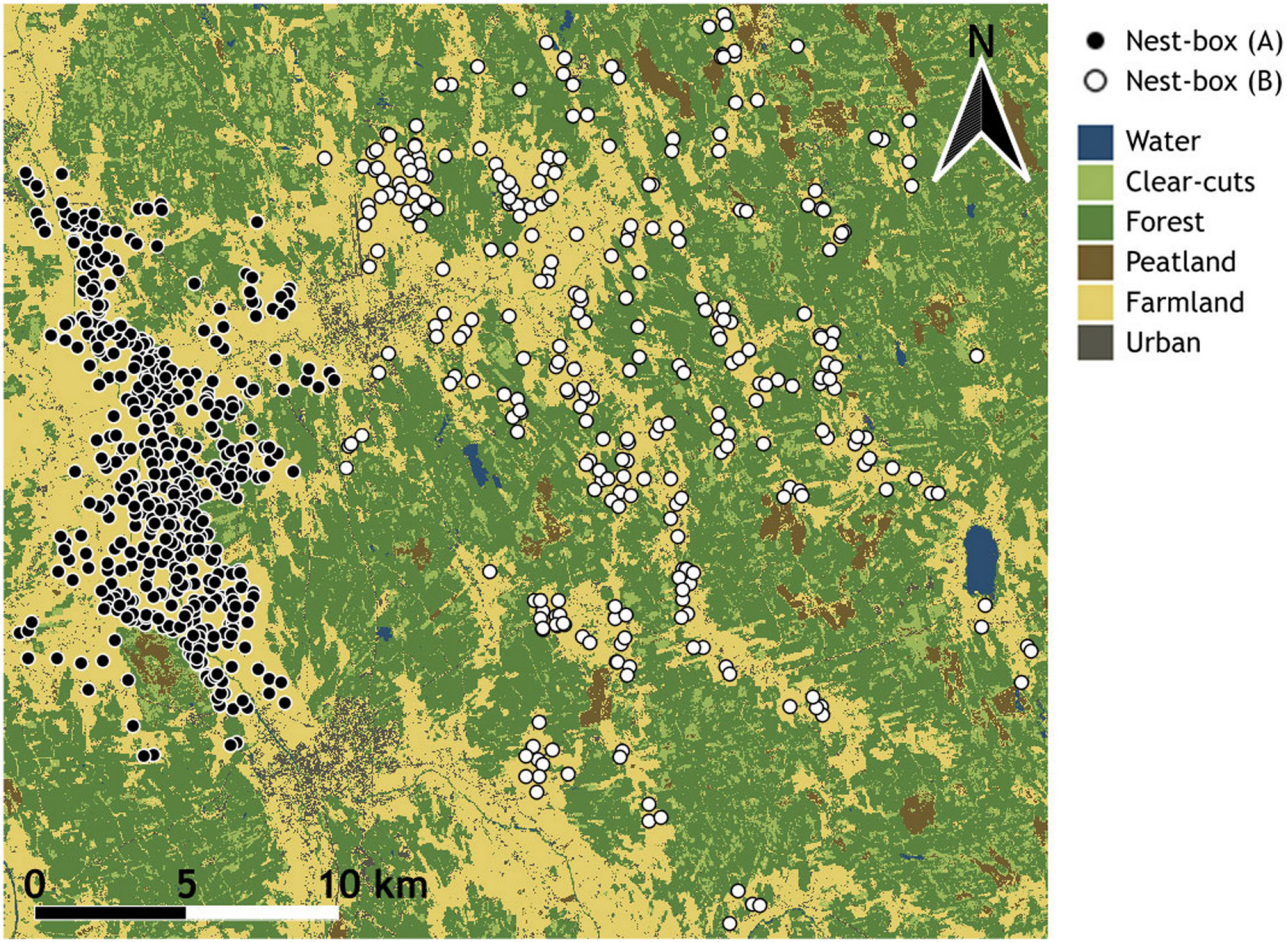
The Kauhava–Lapua study area, showing nest‐boxes of kestrels at the homogeneous (A) and heterogeneous (B) subregion from 1985 to 2021. Land cover types are portrayed according to the classification of Landsat images from 2021 (see “Statistical methods” for further information on land cover classification).

Data were collected from 1985 to 2021 in the municipalities of Kauhava and Lapua, South Ostrobothnia, western Finland. The area is comprised of two subregions: one in the valley of Lapua river (Alajoki field plain) with a largely homogeneous landscape (an area of approximately 150 km^2^; proportion of agricultural fields 50% of the landscape) composed of vast agricultural fields, and one overlapping the municipalities of Kauhava and Lapua (area approximately 250 km^2^; proportion of agricultural fields 20% of the landscape), consisting of smaller (1–10 km^2^) fields alongside with plots of coniferous forest and clear‐cut areas, representing a heterogeneous landscape (Figure 1). In the homogeneous subregion, the average of available nest‐boxes per year was 155.7 (SD = 54.2); in the heterogeneous one, the average was 106.9 (SD = 32.9). On average, 33.7 (SD = 21.7) of these were inhabited per year in the homogeneous subregion, and 31.7 (SD = 19.9) in the heterogeneous one. The number of inhabited nests remained rather uniform in the homogeneous subregion while increased steadily in the heterogeneous one (Figure S1). While the homogeneous region was supplied with a greater number of nest‐boxes, especially during the early years of study, it also encompasses a considerably greater area of suitable kestrel breeding habitat than the heterogeneous region, which consists of proportionally larger expanses of forest unusable to open‐area predators. Therefore, the nest‐boxes in the homogeneous subregion appear more concentrated than the ones in the heterogeneous subregion, in which the preferred habitats (agricultural fields) are more scattered. In reality, there were approximately two nest‐boxes available per every square kilometer of field habitat both at the homogeneous and the heterogeneous region. Overall, the homogeneous subregion is representative of intensive agriculture as it is primarily comprised of vast cereal, potato, and oilseed crop fields with subsurface drainage, whereas the heterogeneous subregion contains additional agricultural plots under less intensive cultivation, such as hayfields, meadows, and open ditches, as well as better access to grassy forest clear‐cuts which open‐country raptors such as kestrels can also use as foraging habitats (Korpimäki, 1986).

Breeding kestrels were captured from nest‐boxes with swing‐door traps in the mid‐nestling period, when the females are stationed at their nests almost invariably and males occasionally during feeding visits (see Terraube et al., 2015; Vasko et al., 2011 for further details on methods). Trapping and ringing of kestrels were executed by Finnish and EU Laws and regulations and under the ringing license (no. 524 to EK) by the Ringing Centre of the Finnish Museum of the Natural History. Ethical approval from the ethics committee for involving animals in this study was not required. All applicable international, national, and/or institutional guidelines for the use of animals were followed.

Captured parent kestrels were sexed and aged based on their plumage (2nd‐year and older individuals; Forsman, 1999), ringed or identified by a pre‐existing ring. Nests were visited routinely to determine the clutch size, and the number of hatchlings and fledglings (see Korpimäki & Wiehn, 1998 for further details). If the clutch was not complete during the first visit to the nest, a two‐day egg‐laying interval was used to estimate the laying date of the first egg (Piechocki, 1982; Village, 1990). However, the vast majority of laying dates of the first eggs were estimated by back‐dating from hatching dates using a 30‐day incubation period and assuming that female kestrels start to incubate from the third laid egg onward (Village, 1990). In total, 2422 nesting attempts were recorded, 1381 of which included a successful capture of a parent male and 1542 the capture of parent females; ring IDs of both parents were recorded from 1285 nests.

To estimate the local abundance of voles, snap traps were used to catch small mammals at 3–5 sites in both subregions of the study area biannually (during mid‐May and mid‐September), totaling, 200–600 trap nights per site in one season (Korpimäki et al., 2005). Vole indices were obtained by dividing the total number of voles (including the species from genera *Myodes* and *Microtus*) caught by hundred trap nights. For linear mixed‐effects models (see below), springtime (mid‐May) vole indices were used for the best estimation of food availability during the breeding season of kestrels. For further details on methodology concerning vole indices, see Korpimäki et al. (2005) and Kujala et al. (2022).

For estimating the relationship between the weather variables and clutch size, the accumulated sums of rainfall (mm) were computed from the 35‐day period preceding the yearly average of the first egg's laying date, reflecting the mean duration of courtship in kestrels (Village, 1990). Correspondingly, for estimating the influence of rainfall on the number of produced fledglings, a time frame of 64 days beginning at the average lay date was chosen to represent the average temperature and the total amount of precipitation during the period of egg‐laying and incubation (29–37 days; Village, 1990) and the nestling stage (27–32 days; Cramp & Simmons, 1980).

The months of April and May were chosen as the timespan for the weather variables to predict the proportion of occupied nest‐boxes, representing the time of arrival and settlement of migrant kestrels to the area (Palokangas et al., 1992). These weather variables consist of mean snow depth (cm) and the sum of precipitation for the two‐month time frame; temperature was initially selected as a predictor variable but omitted from models concerning occupancy rates as it correlated strongly (*r* = −.53) with snow depth while being the less significant of the two variables in predicting occupancy. For predicting the clutch size and the number of fledglings, snow depth and temperature were considered as explanatory variables but discarded from the final model selection because there emerged an association between poor reproductive output and low snow depth and high temperature, which was likely caused by the effects of seasonal decline in clutch size (Korpimäki & Wiehn, 1998; Verhulst et al., 1995). Weather variables are presented as a single value (sum for precipitation, mean for snow depth) per the pre‐defined periods (see above) and based on daily observations by the Finnish Meteorological Institute (https://www.ilmatieteenlaitos.fi/), recorded in the municipality of Kauhava.

### 2.2 Land cover data generation

For estimating territory land cover heterogeneity (hereafter TLCH), yearly land cover data was created by classifying Landsat images provided by the U.S. Geological Survey in resolution of 30 × 30 m with supervised classification using the Semi‐Automated Classification plugin (Congedo, 2016) in QGIS 3.26.1 (QGIS Development Team, 2022). Six classes of land cover types were defined: (1) water (open water bodies), (2) clear‐cut or shrub (recently cut forest or early young‐growth forest), (3) forest (old‐growth forest and late young‐growth forest), (4) peatland (mires, bogs, and peat extraction sites), (5) agricultural field (arable land or other low‐grassy vegetation), and (6) urban (built environment). When creating regions of interest, growing stock volume (i.e. volume of living trees with a diameter of 10 cm or more at breast height, excluding branches) data by Natural Resources Institute Finland (https://kartta.luke.fi/index‐en.html) were used as a guideline for distinguishing forests from clear‐cuts, with clear‐cuts being defined as holding less than 50 m^3^/ha of wood volume. In the 37‐year span of the study, there were 10 years for which no Landsat images without obscuring clouds were available during the snowless season (May–September). For nesting attempts taking place during these years, TLCH was computed from a land cover map from an adjacent year, excluding the year 2001 for which TLCH indices were approximated as an average of values extracted from maps of 1999 and 2003 due to both 2000 and 2002 lacking individually classified land cover maps.

Simpson's diversity index (Simpson, 1949) was computed with the LecoS plugin (Jung, 2016) in QGIS to determine TLCH for each kestrel nest (territory) based on the abundance and number of different land cover types. Territories for individual nests were determined to span the area ranging from the nest to the extent of a 1 km radius, reflecting the distance within which breeding male kestrels forage 90% of the time (Village, 1990).

### 2.3 Statistical methods

Linear mixed‐effects models estimating the yearly proportion of occupied nest sites (nest‐boxes), clutch size, and the number of fledged offspring were computed and ranked in RStudio operating on version 4.2.2 of R (R Core Team, 2022; RStudio Team, 2020) with packages *lme4* (Bates et al., 2015) and *MuMIn* (Bartón, 2023). The proportion of occupied nest sites was modeled with generalized linear mixed‐effects models (GLMMs) following binomial distribution, with the total number of nest‐boxes as the prior weight, whereas clutch size and the number of fledglings were modeled using linear mixed‐effects models (LMMs) with Gaussian distribution. To compare the relative support of competing models, Akaike information criterion (AIC) was applied to each individual model with clutch size or the number of fledglings as the response variable; for models concerning the proportion of occupied nest sites, Akaike information criterion corrected for small sample sizes (AIC_c_) was used.

The model selection began with determining the random effect structure as according to the protocol by Zuur et al. (2009). Then, a saturated model with all potential predictor variables and their interactions was fit, based on which all potential candidate models were formed and ranked by their AIC or AIC_c_ value. Out of the best candidate models (∆AIC or ∆AIC_c_ < 2), we chose the model containing the fewest predictor variables as the final model after confirming that the inclusion of any other predictor featured in the other best candidate models would not improve the model fit based on likelihood‐ratio tests. This way, we were left with the most parsimonious model. See supporting information (Tables S1–S3) for a complete listing of best candidate models for each response variable.

Model averaging approach was considered but not implemented: while pair‐wise correlations between the predictor variables were generally low (ǀ*r*ǀ < .4), we concluded that the presence of multiple weak correlations would hamper meaningful interpretation of model‐averaged parameter estimates (see also Cade, 2015). Multicollinearity was tested in each saturated model with package *mctest* (Imdad & Aslam, 2020). All predictor variables were mean‐centered and scaled to improve the comparability and interpretability of their coefficient estimates (Schielzeth, 2010). Model residuals were both visually evaluated and tested for deviations from normal distribution and spatial autocorrelation, with Moran's *I* test, using package *DHARMa* (Hartig, 2022). For clutch size models, the distribution of residuals simulated with *DHARMa* had a significant (*p* < .001) deviation from the expected distribution according to the Kolmogorov–Smirnov test; however, by visually inspecting the residual plot, no alarming deviations or patterns in residual distribution was found. Q‐Q plot of the final clutch size model is available in the supplementary files (Figure S2). No other deviations from model assumptions were detected (see also below).

Three response variables were chosen for modeling the fluctuations in population parameters: the yearly proportion of nest site occupancy, clutch size in individual breeding attempts, and the number of fledged young in individual breeding attempts. Each response variable was modeled separately with four predictor variables (Table 1) and any potential combination of interaction terms between them. The year was assigned as a random effect with all the response variables, and for models predicting clutch size the ID (ring number) of the female parent was added as random effects to account for the dependency of successive nesting attempts of the same individuals; male ID was not significant as a random effect for predicting either clutch size or the number of fledglings, and the random effect of female ID was not significant in models predicting the number of fledglings. To control for spatial autocorrelation, the study area was divided into blocks using a 2 km × 2 km grid (total of 106 blocks), and block ID was assigned as a random effect for models concerning the number of fledglings; for clutch size models, spatial autocorrelation correction was not necessary and block ID was not included as a random effect due to its insignificance in the saturated model.

**TABLE 1:** The array of predictor variables is considered for predicting the proportion of occupied nest‐boxes, clutch size, and the number of fledglings. In addition, all potential interaction terms between the included predictor variables were considered in the model selection process.

Predictor variable	Proportion of occupancy	Clutch size	Number of fledglings
TLCH index		x	x
Subregion	x		
Mean snow depth (cm)	x		
The sum of rainfall (mm)	x	x	x
Vole index	x	x	x
Male age		x	x
Total no. of candidate models	113	113	113

For models predicting clutch size or the number of fledglings, the TLCH (Simpson's) index was used as a predictor variable indicating the degree of habitat heterogeneity. For estimating the proportion of occupancy, subregion (two levels: A = Alajoki, with a more homogeneous landscape, or B = Kauhava, with a more heterogeneous landscape) was used instead as a general indicator of discrepancy in habitat composition, because TLCH indices of kestrel nests had a significant difference between the two regions (mean 0.29 in the homogeneous region and 0.54 in the heterogeneous one; two‐sample *t*‐test, *t* = −40.3, *p* < .001). Abundance indices of voles in mid‐May were used as predictor variables indicating the overall food availability during the breeding season. Indices were obtained by dividing the total number of voles caught with snap traps by a hundred trap nights. In general, vole indices were slightly greater at the heterogeneous (mean 5.0, SD = 5.0) than at the homogeneous (mean 4.3, SD = 4.1) subregion, although this difference did not reach statistical significance (Wilcoxon signed‐rank test, *z* = −1.9, *p* = .057).

In addition to the TLCH index, vole index, and weather variables, we chose to include the age of male parent (two levels: 2nd‐year or older) as a predictor variable (Table 1) to our models of clutch size and fledgling production to increase the predictive capacity, as older males have consistently higher rates of food provisioning, reflected both in the initial clutch size and subsequently in the final number of fledglings (Korpimäki & Wiehn, 1998; Palokangas et al., 1992). The initiation date of egg‐laying (lay date) was considered as an additional predictor variable as well, as it represents the settling order of nest sites and territories and has been shown to have a strong negative correlation with breeding success (Korpimäki & Wiehn, 1998; Village, 1985), but ultimately left out to avoid it masking other effects associated with territory quality. The age of the female parent was not considered a predictor variable, as it has been shown to have no obvious effect on clutch size in kestrels (Korpimäki & Wiehn, 1998). For models concerning the number of fledglings, nesting attempts with zero fledglings were excluded (58 out of 1285 nests, 4.5%), as total nest failure was likely caused by predation, which the chosen random effects and predictor variables are presumably unsuitable to control or predict. After the removal of total nest failures, the average sample size per year was 33 nesting attempts.

## 3 RESULTS

### 3.1 Breeding density

**FIGURE 2:**
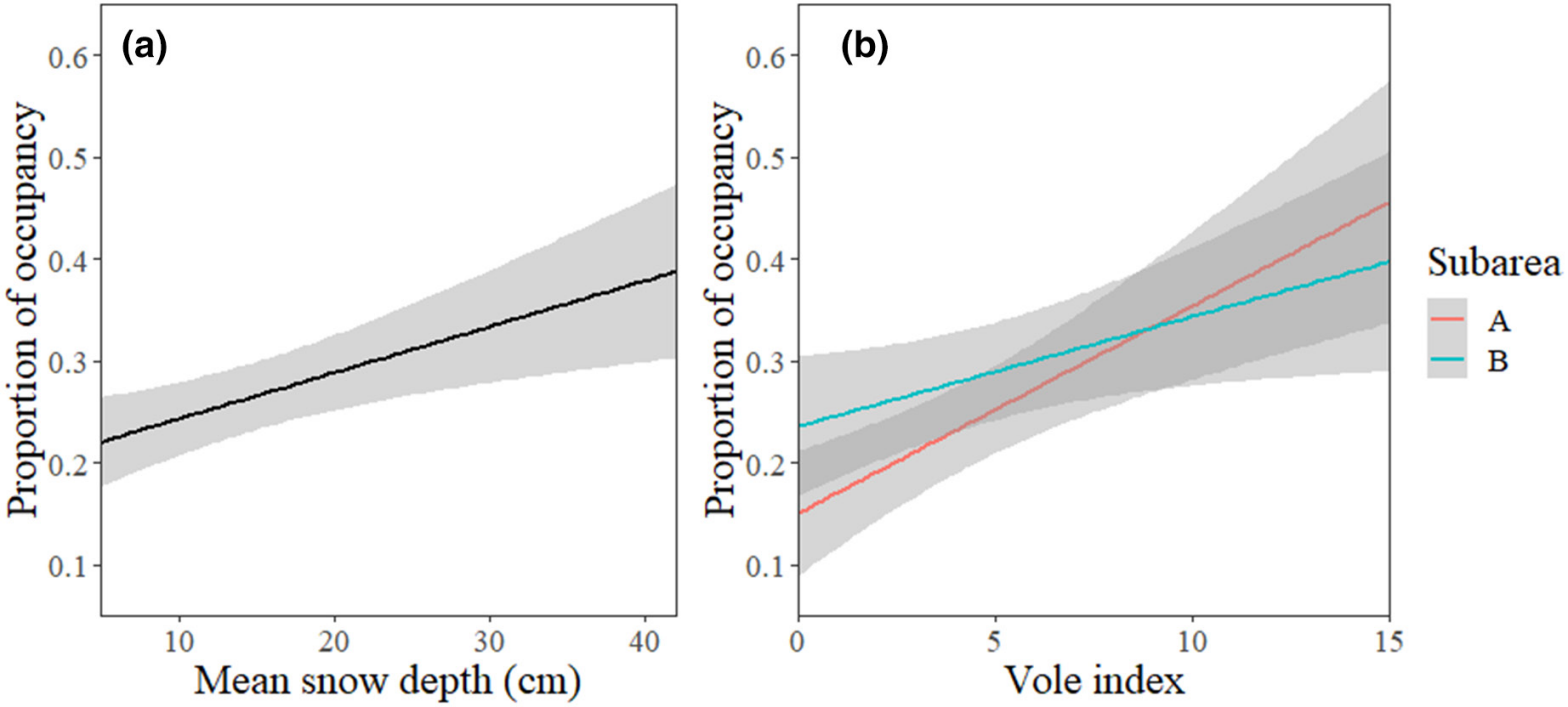
The proportion of occupied nest‐boxes of kestrels in relation to (a) the average snow depths of April–May and (b) the springtime vole indices (voles per 100 trap nights). Effect sizes are shown with 95% CIs. In (b), the interaction between the proportion of occupancy of kestrel nest‐boxes and vole indices are presented separately for the homogeneous (A) and the heterogeneous (B) subarea.

Considering the proportion of occupied nest‐boxes as a response variable, the most parsimonious model included snow depth, vole index, subregion, and the interaction of subregion and vole index as explanatory variables (Table 2). The breeding density of kestrels was positively associated with the abundance of voles and the depth of the snow cover during the time of settlement in spring (Figure 2). Overall, the nest sites in the heterogeneous subregion were occupied in higher proportions (mean 29.0%, SD = 0.15) than in the more homogeneous area (mean 24.3%, SD = 0.16), although high vole availability appeared to amplify the occupancy rates more at the homogeneous subregion (Figure 2b).

**TABLE 2:** Estimated parameters of variables predicting the proportion of occupied nest sites in the Eurasian kestrel in a generalized linear mixed‐effects model, presented alongside their SEs, 95% confidence intervals, and *z*‐ and *p*‐values.

Effect	Estimate	SE	*z*	*p*	95% CI
Snow depth (cm)	0.25	0.11	2.25	.024	(0.033, 0.48)
Subarea (B)	0.28	0.053	5.26	1.4 × 10^−7^	(0.18, 0.38)
Vole index	0.37	0.070	5.32	1.1 × 10^−7^	(0.24, 0.51)
Subarea × vole index	−0.27	0.056	−4.80	1.6 × 10^−6^	(−0.38, −0.16)
Intercept	−1.3	0.11	*—*	*—*	(−1.5, −1.0)

*Note*: The proportion of occupied nest sites (*n* = 74, *R* ^2^ conditional = .16, AIC_c_ = 570.7, random effects: year).

### 3.2 Clutch size

**FIGURE 3:**
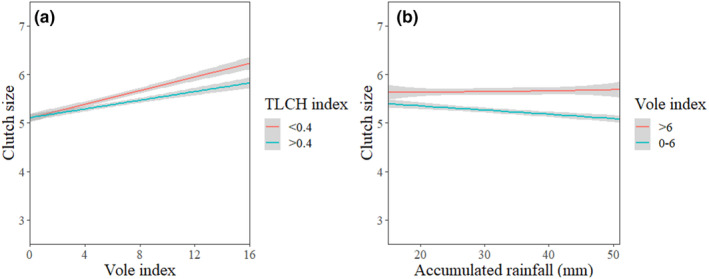
Clutch size of kestrels in relation to vole abundance in territories with low (TLCH <0.4) and high (TLCH >0.4) landscape heterogeneity (a) as well as in relation to the total accumulation of rainfall during the average courtship period (b) under both high (vole index >6) and low (vole index <6) food availability. Effect sizes are shown with 95% CIs.

For estimating clutch size, the most parsimonious model contained TLCH, rainfall, male age, vole index, and the interactions of rain and vole index, TLCH and vole index, and rain and male age (Table 3). The vole index had a positive effect on clutch size, whereas TLCH and male age were negatively associated with it; furthermore, the reductive effect of TLCH was greater in high vole abundance. Independently, rainfall did not appear to influence the clutch size, but it had significant interactions both with the vole index and with male age. High vole abundance appeared to downplay the diminishing effects of precipitation on clutch size (Figure 3).

**TABLE 3:** Estimated parameters of variables predicting clutch size in the Eurasian kestrel in a linear mixed‐effects model, presented alongside their SEs, 95% confidence intervals, and *t*‐ and *p*‐values.

Effect	Estimate	SE	*t*	*p*	95% CI
TLCH	−0.080	0.024	−3.39	7.3 × 10^−4^	(−0.13, −0.034)
Vole index	0.18	0.054	3.37	9.8 × 10^−4^	(0.067, 0.29)
Rainfall (mm)	0.050	0.082	0.602	.55	(−0.12, 0.22)
Male age (2nd year)	−0.57	0.084	−6.78	1.9 × 10^−11^	(−0.73, −0.40)
TLCH × vole index	−0.068	0.023	−2.95	.0033	(−0.11, −0.023)
Vole index × rainfall	0.21	0.068	3.16	.0020	(0.081, 0.35)
Rainfall × male age	0.35	0.10	3.37	7.7 × 10^−4^	(0.15, 0.56)
Intercept	5.6	0.077	—	—	(5.4, 5.7)

*Note*: Clutch size (*n* = 1285, *R* ^2^ conditional = .50, AIC = 3140.5, random effects: year, female ID).

### 3.3 Number of fledglings

**FIGURE 4:**
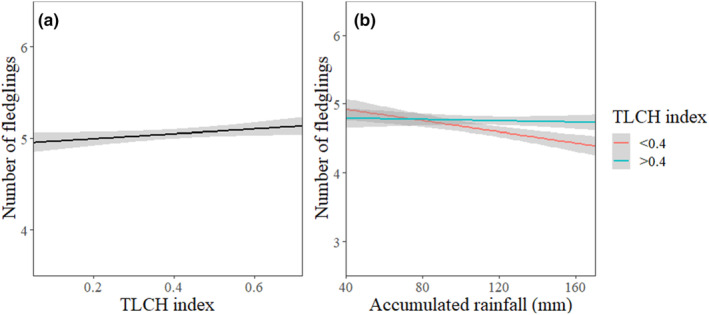
The number of fledglings in kestrel nests in relation to territory land cover heterogeneity (TLCH) (a) as well as the influence of rainfall on the number of fledglings in territories with high (TLCH index >0.4) and low (TLCH index <0.4) landscape heterogeneity (b). Effect sizes are shown with 95% CIs.

**FIGURE 5:**
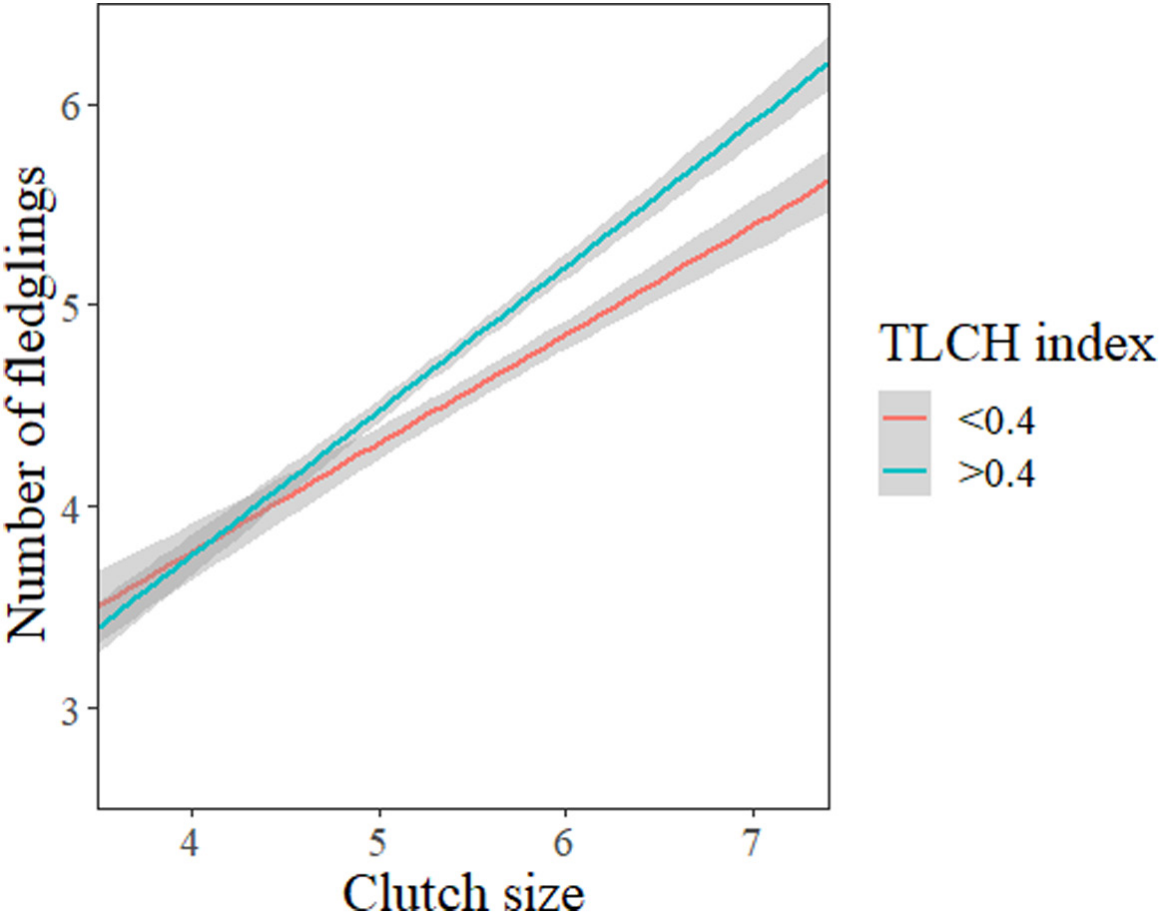
The comparison of initial clutch size and realized number of fledglings in territories with high (TLCH index >0.4) and low (TLCH index <0.4) land cover heterogeneity. Effect sizes are shown with 95% CIs.

When predicting the number of fledglings, the most parsimonious model contained the TLCH index, male age, rainfall, and the interaction of TLCH and rain as predictor variables (Table 4). While fledgling production had a strong negative correlation with male age, it increased slightly alongside TLCH indices (Figure 4a). In addition, high TLCH indices appear to buffer against the detrimental effects of precipitation on the number of fledglings (Figure 4b). Ultimately, the number of fledglings relative to clutch size was greater in habitats with high TLCH and when clutch size was large (Figure 5).

**TABLE 4:** Estimated parameters of variables predicting the number of surviving fledglings in the Eurasian kestrel in a linear mixed‐effects model, presented alongside their SEs, 95% confidence intervals, and *t*‐ and *p*‐values.

Effect	Estimate	SE	*t*	*p*	95% CI
TLCH	0.11	0.046	2.5	.020	(0.0038, 0.20)
Rainfall (mm)	−0.090	0.11	−0.824	.42	(−0.32, 0.13)
Male age (2nd year)	−0.90	0.14	−6.48	1.35 × 10^−10^	(−1.2, −0.62)
TLCH × rainfall	0.12	0.039	3.14	.0018	(0.046, 0.20)
Intercept	4.5	0.12	—	—	(4.2, 4.7)

*Note*: Number of fledglings (*n* = 1212, *R* ^2^ conditional = .23, AIC = 4164.1, random effects: year, block ID).

## 4 DISCUSSION

From this study, we made the following main findings: (1) the number of nest sites occupied by kestrels was influenced by the presence and depth of snow cover as well as abundance of main foods; (2) clutch size in kestrels appears negatively associated with territory land cover heterogeneity but positively affected by vole abundance, particularly in rainy conditions; (3) despite having a negative influence on clutch size, territory land cover heterogeneity had a positive effect on the fledgling production of kestrels, and this advantage appeared greater during seasons of high precipitation. Ultimately, variables predicting large clutches and high numbers of fledglings were not uniform, with the most outstanding difference being the significance of territory land cover heterogeneity regarding realized reproductive output, meaning that the benefits of heterogeneous territories are reaped only after the young have hatched while being insubstantial during territory selection and settlement.

### 4.1 Breeding density

The influence of vole abundance on the density of breeding kestrel populations appears as expected, as adult kestrels, particularly females, and yearlings, are attracted to areas with a plentiful supply of their main prey species (Vasko et al., 2011). Furthermore, the positive effect of long‐lasting snow cover in spring on kestrel breeding density might be linked to the availability of voles. Snow cover protects small mammals from predation, and the early disappearance of snow in spring presents the open‐area dwelling *Microtus* voles as easier targets for both mammalian and avian predators, many of which begin their breeding season earlier than the migratory kestrels (Korpimäki, 1987; Korpimäki & Norrdahl, 1991; Sonerud, 1986). When snow cover persists to late spring, a greater proportion of voles are preserved for kestrels arriving in the area, persuading them to settle. Similar causation was suggested by Sharikov et al. (2019) who reported a corresponding correlation between snow depth and kestrel pair numbers. Both the length of the snowy season and the depth of snow have been declining in the southern and western parts of Finland, whereas a positive trend of snow accumulation has been observed in areas located in the northern and eastern parts of the country (Luomaranta et al., 2019). Increasing spatial variation in the presence of snow cover may therefore induce change in the distribution of appealing habitats.

As homogeneous expanses of agricultural lots are likely to appear more lucrative as foraging grounds than uncropped vegetation during early spring because they are the first to thaw, kestrels may identify high vole abundance more effortlessly at homogeneous territories (Koivusalo & Kokkonen, 2002; Sonerud, 1986). Correspondingly, the heterogeneous area seemed to host a more invariable number of breeding pairs, indicating that the assessed quality of its territories may be less dependent on vole densities. The declining trend of kestrel breeding attempts at the homogeneous subregion could be explained not only by the degraded habitat quality and the decreasing numbers of barns to which nest‐boxes have been attached (at least 36 barns with nest‐boxes were destroyed during the span of the study) but also by the altered pattern of food availability. If the cyclic nature of vole population fluctuations continues to be dampened by climate change (Avotins et al., 2023; Cornulier et al., 2013; Ims et al., 2008; Korpela et al., 2013), years of high breeding densities of kestrels may become more infrequent at environmentally homogeneous areas as the amplitudes of vole cycle peaks diminish.

### 4.2 Clutch size

In our study population, the influence of the abundance of voles and the age of male parent on clutch size were consistent with the findings documented in previous studies (e.g. Korpimäki & Wiehn, 1998; Palokangas et al., 1992). As the largest clutches are generally laid in the early days of the breeding season in kestrels (Korpimäki & Wiehn, 1998), and because the lay date is indicative of the settling order (Palokangas et al., 1992; Village, 1985), they should be laid in the most sought‐after territories according to the IDD model. Notably, territory land cover heterogeneity had a negative influence on clutch size, indicating that during the prospecting and courtship phases of breeding, the heterogeneity of a territory does not elevate its appraised quality.

While rainfall had no significant effect on clutch size by itself, its interactions with vole availability indicate that clutch size is conditional to the plentiful availability of food particularly during rainy seasons. For open‐area predators such as kestrels, heavy rainfall hampers their ability to forage efficiently, leading to reduced provisioning by males which could limit the clutch size (Laux et al., 2016; Redpath et al., 2002).

The interaction between male age and rainfall is most likely explained by the absence of young males breeding during years of high precipitation: only 106 out of 1285 (8.2%) nesting attempts included a 2nd‐year male parent, and none of them took place during the 2 years of heaviest rainfall preceding laying when precipitation sums ranged from 53.5 to 100.8 mm during courtship. It is possible that there is a higher threshold for young males to attempt breeding in regard to disadvantageous environmental conditions or that they are simply unable to forage efficiently enough to maintain steady provision rates in courtship if foraging opportunities are scarcely available. Yearling males of other vole‐subsisting avian predators are also not able to feed their partners so that they can lay eggs in poor environmental conditions (e.g. Laaksonen et al., 2002).

### 4.3 Number of fledglings

Consistent with their influence on clutch size, yearling males are associated with diminished fledgling production. Conversely, despite its slight disadvantage in determining clutch size, territory land cover heterogeneity appears to have a positive effect on the fledgling production of kestrels. Benefits of landscape heterogeneity of territories to reproductive output have been observed from both open‐area raptors (Orozco‐Valor & Grande, 2021; Sumasgutner et al., 2019) and forest‐dwelling species (Byholm et al., 2007). Heterogeneous habitats with mixed vegetation are populated by diverse communities of small mammals and birds, and therefore provide a better assortment of alternative prey whenever preferred species are scarce (Butet et al., 2006), but this diversification of prey does not occur universally; for a Spanish kestrel population, the heterogeneity of habitats was found to be negatively correlated with diet diversity (Navarro‐López & Fargallo, 2015). In our study area, the dominating land cover type of homogeneous kestrel territories is cropland, whereas the Spanish study site comprised mostly of cattle pastures, which are likely hosting more diverse communities of potential prey species compared to crop fields (Cerezo et al., 2011).

As springtime vole indices were not featured in the top model predicting the number of fledged offspring, we conclude that they are not representative of the realized availability of food during the nestling phase of kestrels. Moreover, the discrepancy in the relevance of territory land cover heterogeneity before and after laying could be due small‐scale spatiotemporal variation in the dispersion, abundance, and availability of prey animals: during the arrival and settlement of migrant kestrels, arable fields are still largely undisturbed and capable of hosting dense vole populations, but as the season progresses, farming practices such as plowing, harvesting, and rodenticide use may reduce the survival of *Microtus* voles in areas without uncropped vegetation available for shelter (Ivanter et al., 2013; Jacob et al., 2013).

The disadvantageous effects of heavy rainfall on breeding output and nestling survival have been consistently observed among various populations and species of raptors (Anctil et al., 2014; Caravaggi et al., 2019; Mearns & Newton, 1988; Redpath et al., 2002; Rodríguez & Bustamante, 2003; Selås, 2001). As our study population breeds almost exclusively in nest‐boxes which shelter nestlings from direct exposure to heavy rain, we believe that precipitation‐related mortality was caused primarily by starvation instead of hypothermia (Anctil et al., 2014; Kostrzewa & Kostrzewa, 1990). Rainfall limits the availability of foraging opportunities and restricts the mobility of raptors who typically hover, soar, or perch without cover when searching for prey, reducing the rates of prey deliveries to nests (Dawson & Bortolotti, 2000; Newton, 1978; Rijnsdorp et al., 1981; Village, 1990). While the total amount of rainfall during the nestling period had a deteriorating effect on fledgling survival, territory land cover heterogeneity appeared to buffer against the effects of rain. As higher provision rates translate to higher numbers of surviving fledglings (Tolonen & Korpimäki, 1994; Wiehn & Korpimäki, 1997), we surmise that overall, heterogeneous habitats provide better circumstances for foraging during the nestling phase, allowing parent kestrels to compensate more efficiently for the loss in energy intake of nestlings resulting from bouts of rainy weather. As climate change is expected to increase summertime precipitation in Finland (Ylhäisi et al., 2010), the threshold for foraging habitats viable to feed large broods may grow higher as well.

## 5 CONCLUSIONS

The independent and interactive effects of weather conditions and territory landscape heterogeneity on kestrel breeding performance suggest that climate change and the intensification of agricultural practices have an influence over kestrel population fluctuations. The density of breeding kestrel populations is primarily governed by vole abundance and springtime snow depth, both of which are susceptible to changes induced by climate warming. While heterogeneous territories are not initially preferred over homogeneous ones, heterogeneous habitats may provide better circumstances for foraging during the nestling period, which ensures nestling survival, particularly during adverse weather conditions. Therefore, breeding in areas under intense agricultural use is more suboptimal to kestrels than their territory preferences would imply, which suggests that the homogeneous landscape developed by the intensification of agriculture creates an ecological trap for kestrels. Even though the kestrel population in Finland remains currently in a state of growth, this increase has already decelerated, and the trend may turn to a decline as climate change and the intensification of agricultural practices advance, following the fate of numerous other farmland species.

## AUTHOR CONTRIBUTIONS

**Inga Kujala:** Conceptualization (supporting); data curation (supporting); formal analysis (lead); funding acquisition (equal); methodology (lead); resources (equal); software (lead); visualization (lead); writing – original draft (lead); writing – review and editing (supporting). **Hannu Pöysä:** Conceptualization (equal); methodology (supporting); supervision (equal); validation (equal); writing – review and editing (equal). **Erkki Korpimäki:** Conceptualization (lead); data curation (equal); funding acquisition (equal); investigation (lead); methodology (supporting); project administration (lead); supervision (equal); validation (equal); writing – review and editing (equal).

## CONFLICT OF INTEREST STATEMENT

The authors declare no conflict of interest.

## Supporting information


Appendix S1.

Appendix S2.

## Data Availability

The data that supports the findings of this study are available in the Appendix S2 of this article.
