# Supplementary material for: Interactive effects of agricultural landscape heterogeneity and weather conditions on breeding density and reproductive success of a diurnal raptor
Source: Ecol Evol. 2024 Mar 12;14(3):e11155. doi: 10.1002/ece3.11155 (PMC10932691; doi:10.1002/ece3.11155)
Supplement: Supplementary file 1 — Appendix S1. [file ECE3-14-e11155-s001.docx]

**Supporting Information**


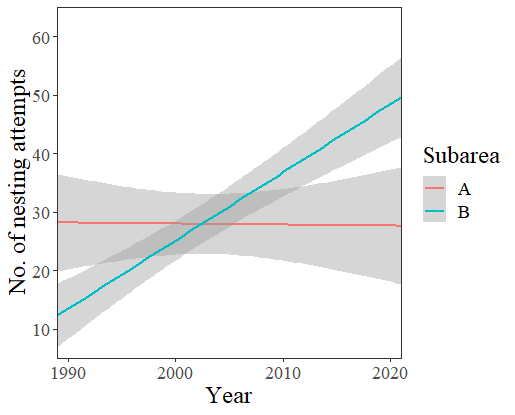


**Figure S1.** The trend of nesting attempts of kestrels at the subregion with more homogenous landscape (A) and the subregion with more heterogenous landscape (B) in the Kauhava-Lapua study site from 1985 to 2021, with 95% confidence intervals.


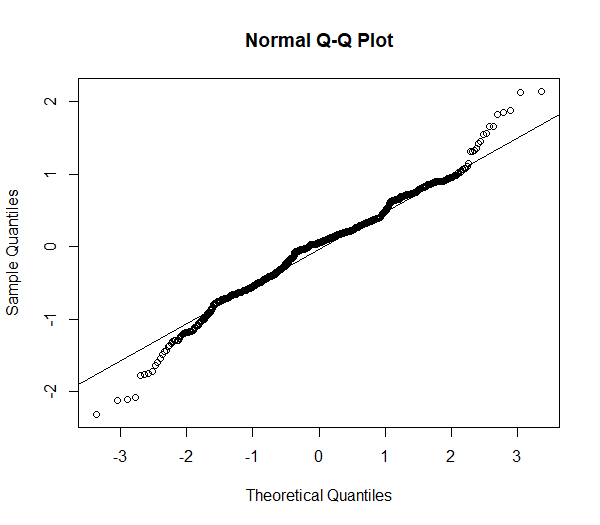


**Figure S2.** Plotted Q-Q plot depicting observed values of clutch size in relation values predicted by the final model predicting clutch size in the Eurasian kestrel (*n* = 1 285).

**Table S1.** Parameter estimates of top candidate models (∆ AIC_c_ < 2) predicting the proportion of occupied nest sites in the Eurasian kestrel alongside their degrees of freedom, AIC_c_ values, and ∆ AIC_c_ in reference to the model with smallest AIC_c_. Interaction variables not featured in any of the top models are absent from the table. The model with least predictor variables is shown in **bold.**

| *Intercept* | rainfall (mm) | snow depth (cm) | subarea (B) | vole index | rainfall x snow depth | snow depth x subarea | snow depth x vole index | subarea x vole index | df | AIC_c_ | ∆ AIC_c_ |
| --- | --- | --- | --- | --- | --- | --- | --- | --- | --- | --- | --- |
| -1.3 | – | 0.29 | 0.28 | 0.42 | – | -0.11 | – | -0.24 | 7 | 569.5 | 0 |
| **-1.3** | **–** | **0.25** | **0.28** | **0.37** | **–** | **–** | **–** | **-0.27** | **6** | **570.7** | **1.2** |
| -1.3 | – | 0.29 | 0.31 | 0.37 | – | – | -0.089 | -0.22 | 7 | 570.7 | 1.2 |
| -1.3 | 0.015 | 0.29 | 0.28 | 0.43 | -0.27 | -0.11 | – | -0.25 | 9 | 571.2 | 1.6 |
| -1.2 | 0.013 | 0.30 | 0.31 | 0.38 | -0.31 | – | -0.11 | -0.22 | 9 | 571.4 | 1.9 |

**Table S2**. Parameter estimates of top candidate models (∆ AIC < 2) predicting clutch size in the Eurasian kestrel alongside their degrees of freedom, AIC values, and ∆ AIC in reference to the model with smallest AIC. The model with least predictor variables is shown in **bold**.

| *Intercept* | rainfall (mm) | male age (2^nd^ year) | TLCH | vole index | rainfall x male age | rainfall x TLCH | rainfall x vole index | male age x TLCH | male age x vole index | TLCH x vole index | df | AIC | ∆ AIC |
| --- | --- | --- | --- | --- | --- | --- | --- | --- | --- | --- | --- | --- | --- |
| 5.6 | 0.036 | -0.57 | -0.071 | 0.17 | 0.36 | 0.037 | 0.21 | -0.12 | – | -0.056 | 13 | 3139.9 | 0 |
| 5.6 | 0.032 | -0.57 | -0.08 | 0.17 | 0.36 | 0.04 | 0.21 | – | – | -0.055 | 12 | 3140 | 0.067 |
| 5.6 | 0.053 | -0.57 | -0.07 | 0.18 | 0.36 | – | 0.22 | -0.13 | – | -0.068 | 12 | 3140.1 | 0.15 |
| **5.6** | **0.050** | **-0.57** | **-0.080** | **0.18** | **0.35** | – | **0.21** | – | – | **-0.068** | **11** | **3140.5** | **0.58** |
| 5.6 | 0.031 | -0.57 | -0.079 | 0.17 | 0.36 | 0.040 | 0.21 | – | 0.034 | -0.054 | 13 | 3141.8 | 1.9 |
| 5.6 | 0.035 | -0.57 | -0.071 | 0.17 | 0.37 | 0.037 | 0.21 | -0.12 | 0.013 | -0.056 | 14 | 3141.9 | 2 |

**Table S3.** Parameter estimates of top candidate models (∆ AIC < 2) predicting the number of fledglings in the Eurasian kestrel alongside their degrees of freedom, AIC values, and ∆ AIC in reference to the model with smallest AIC. Interaction variables not featured in any of the top models are absent from the table. The model with least predictor variables is shown in **bold**.

| *Intercept* | male age (2^nd^ year) | rainfall (mm) | TLCH | vole index | male age x rainfall | male age x TLCH | rainfall  x TLCH | rainfall x vole index | df | AIC | ∆ AIC |
| --- | --- | --- | --- | --- | --- | --- | --- | --- | --- | --- | --- |
| ***4.5*** | **-0.90** | **-0.091** | **0.11** | **–** | **–** | **–** | **0.12** | **–** | **8** | **4164.1** | **0** |
| *4.5* | -0.90 | -0.072 | 0.10 | 0.12 | – | – | 0.11 | 0.14 | 10 | 4164.8 | 0.77 |
| *4.5* | -0.90 | -0.092 | 0.12 | – | – | -0.14 | 0.12 | – | 9 | 4165.1 | 1.1 |
| *4.5* | -0.89 | -0.084 | 0.11 | 0.062 | – | – | 0.12 | – | 9 | 4165.5 | 1.4 |
| *4.5* | -0.90 | -0.074 | 0.11 | 0.12 | – | -0.14 | 0.11 | 0.15 | 11 | 4165.9 | 1.8 |
| *4.5* | -0.90 | -0.090 | 0.11 | – | -0.0099 | – | 0.12 | – | 9 | 4166.0 | 1.9 |
